# Increased Serum CD14 Level Is Associated with Depletion of TNF-α in Monocytes in Migraine Patients during Interictal Period

**DOI:** 10.3390/ijms18020398

**Published:** 2017-02-13

**Authors:** Slawomir Michalak, Alicja Kalinowska-Lyszczarz, Danuta Wegrzyn, Adam Niezgoda, Jacek Losy, Krystyna Osztynowicz, Wojciech Kozubski

**Affiliations:** 1 Department of Neurochemistry and Neuropathology, Chair of Neurology, Poznan University of Medical Sciences (PUMS), Poznan 60-355, Poland; akalinowskalyszczarz@ump.edu.pl (A.K.-L.); osztynowiczkr@ump.edu.pl (K.O.); 2 Chair of Neurology, PUMS, Poznan 60-355, Poland; neurosk@ump.edu.pl (D.W.); adamniezgoda@wp.pl (A.N.); wkozubski@ump.edu.pl (W.K.); 3 Department of Clinical Neuroimmunology, Chair of Neurology, PUMS, Poznan 60-355, Poland; jlosy@ump.edu.pl

**Keywords:** migraine, monocytes, tumor necrosis factor alpha (TNF-α), macrophage inflammatory protein-1 (MIP-1), CD-14

## Abstract

The aim of the present study was to investigate the levels of circulating CD14 in relation to the expression of tumor necrosis factor alpha (TNF-α) in monocytes, and serum levels of TNF-α and macrophage inflammatory protein-1 (MIP-1) in migraine patients. Numerous studies revealed controversial changes in the components of the immune system during attacks and the interictal period in migraine patients. Our study included 40 migraineurs and 39 controls. The levels of TNF-α, MIP-1 and CD14 were measured in peripheral monocytes and in sera with the Enzyme-Linked Immunosorbent Assay (ELISA) method, and the monocyte expression of TNF-α was also analysed by immunostaining. Serum CD14 concentrations were higher and the expression of TNF-α in monocytes was decreased in migraineurs. The serum MIP-1 level correlated with Verbal Rating Scale (VRS); the MIP-1:CD14 ratio in monocytes correlated with Visual Analogue Scale (VAS); the MIP-1:CD14 ratio correlated with Migraine Severity (MIGSEV)-Pain scores; and serum CD14 concentration correlated with migraine duration in years. Increased serum CD14 and depletion of TNF-α in monocytes can orchestrate other components of the immune system during the interictal period.

## 1. Introduction

The immune system and inflammatory mediators have been implicated in migraine pathophysiology, although migraine is not recognized as a classically inflammatory disorder. The results of numerous clinical and experimental studies support the neurogenic inflammation theory of migraine pathophysiology. Research in this area was inspired by clinical observation of comorbidity of migraine and atopic or inflammatory diseases [1,2,3,4]. Clinical data obviously need more detailed research at the molecular level. A number of studies have been published exploring the role of inflammation during migraine attacks and in the interictal phase.

Attacks of migraine have been associated with a variety of changes in the immune system, including complement components, immunoglobulin levels, cytokine concentrations and lymphocyte subtypes count [5,6,7,8,9,10,11,12,13,14,15].

The interictal phase was characterized by various changes in immunoglobulin levels [16,17] and lymphocyte counts [7,8,11,18], decreased monocyte chemotactic response [10] and decreased monocytic β-endorphin levels [19]. Interictal circulating cytokines abnormalities included increased serum levels of interleukin 1 alpha (IL-1α), interleukin 1 beta (IL-1β), tumor necrosis factor alpha (TNF-α) [13,14] and decreased interleukin 2 (IL-2) [20].

The presence of neurogenic inflammation in the trigeminovascular system during migraine attack [21] emphasizes the importance of inflammatory mediators; however, there is no conclusive evidence. Cytokine secretion in migraine patients is triggered by calcitonin gene-related peptide (CGRP) [22]. On the other hand, TNF-α, IL-1, interleukin 6 (IL-6) and interleukin 8 (IL-8) may promote hyperalgesia—a clinically important symptom during migraine attack [23]. These observations provide evidence for the use of non-steroid anti-inflammatory drugs (NSAIDs) for migraine attack treatment.

Increased expression of IL-1β in the dura mater, further mast cell degranulation and IL-6 production stimulates inducible nitric oxide (NO) synthase (iNOS) in meningeal macrophages [24,25].

Mast cells, localized predominantly in the meninges and activated by the substance P-depending process, were also suggested to play a role in migraine pathophysiology [26]. Migraine precipitating factors, such as stress and sex hormones, may also trigger meningeal mast cells activation [27,28] and the subsequent release of inflammatory mediators, activating the trigeminovascular system.

Monocytes are immune cells that are suggested to play a role in migraine pathophysiology. Increased NF-κB activity in monocytes from internal jugular blood of migraine patients peaked 2 h after attack onset and was accompanied by a transient reduction in IκBα expression. Up-regulation of monocytic iNOS was observed at 4 h, maintained at 6 h and reduced at the end of the attack [29].

The purpose of the present study was to investigate the role of CD14 in relation to the expression of TNF-α in monocytes, serum levels of TNF-α and MIP-1 Macrophage Inflammatory Protein-1 (MIP-1) in migraine patients during the interictal period.

## 2. Results and Discussion

### 2.1. Results

The clinimetric evaluation of migraineurs included in the study revealed moderate to severe intensity of migraine attacks. This was demonstrated by mean results of MIDAS scale (43 ± 18), MIGSEV: MIGSEV-Pain (3.48 ± 0.51), MIGSEV-Nausea (3.24 ± 0.66), MIGSEV-Activity (3.40 ± 0.58), MIGSEV-Tolerability (2.72 ± 0.46), VAS scale (8 ± 2) and VRS (3.0 ± 1.5).

We have found lower white blood cells (WBC) count in migraine patients (*p* = 0.04) and a higher level of anticardiolipin IgG compared to the controls (*p* = 0.04), otherwise no significant differences in the metabolic and inflammatory profile were found, see Table 1 and Table 2.

We have found a significantly higher concentration of soluble CD14 in migraineurs’ sera (*p* = 0.009). TNF-α expression in monocytes from migraine patients was decreased compared to controls (*p* < 0.001). There were no statistically significant differences in serum concentrations of MIP-1 and TNF-α as well as in MIP-1:CD14 and TNF-α:CD14 ratios in monocytes between migraine patients and controls, see Table 3.

The expression of TNF-α in monocytes did not differ between migraineurs with and without aura (*p* = 0.27), however, in both groups of migraineurs, macrophage TNF-α expression was significantly (*p* < 0.001) lower than in controls.

The comparison of TNF-α, MIP-1, CD14 and MIP-1:CD14 and TNF-α:CD14 ratios did not show differences between migraineurs with and without aura, see Table 4.

TNF-α expression in monocytes did not differ between migraineurs using NSAIDs as abortive treatment and those not using NSAIDs (*p* = 0.44). None of the studied parameters were influenced by NSAID treatment, see Table 5.

The correlations between analyzed chemokines, cytokines and results of routine laboratory tests are presented in Table 6 and correlations with clinimetric measures are shown in Table 7.

### 2.2. Discussion

Monocytes are immune system components that are involved in migraine pathomechanism, as evidenced in studies that have shown similar, as described by us, abnormalities in their function. Abortive treatment for migraine attacks may have an effect on monocytes’ function. Chemotaxis of monocytes is inhibited by acetylsalicylic acid, while metoprolol, metoclopramide, dihydroergotamine and sumatriptan did not show such effect [30]. Furthermore, 5HT receptor expression increases after sumatriptan administration, probably as a result of 5HT turnover activation and increased availability of 5HT displaced by this triptan from cerebrovascular receptors [31]. Nitric oxide production and prostaglandin E2 release by peripheral monocytes are increased in migraine patients [32]. Stimulation of nitric oxide synthase and cyclooxygenase was thus suggested in monocytes even during the interictal period.

In the present study, we have found increased serum CD14 concentration. CD14 is a myeloid cell-surface receptor and a soluble plasma protein (sCD14), which binds lipopolysaccharide (LPS) [33,34]. Serum concentration of CD14 is increased during the systemic response to infection and mediates production of proinflammatory cytokines (TNF-α, IL-1, IL-6) [35]. CD14 causes increased release of LPS from the monocyte and its binding to plasma lipoproteins. This leads further to the reduction in the ability of monocytes to produce cytokines in response to LPS [36]. sCD14 promotes phospholipid efflux from cells [37] and it originates from monocytes [38]. Intracerebral administration of rsCD14 in animal experiments caused increased cytokine levels in the cerebrospinal fluid [39] and indicates a proinflammatory effect of CD14 in the central nervous system. Thus, we may hypothesize that a higher level of CD14 is related to the pathomechanism involved in monocytes stimulation in migraine patients. This may subsequently lead to the decreased expression of TNF-α in monocytes, which was observed in our study. Depletion of TNF-α in monocytes of migraine patients seems to be the result of prolonged stimulation of peripheral monocytes. The role of CD14 in this phenomenon is indicated by the positive correlation of its serum levels and duration of migraine that we have revealed. Even if we have not found significant differences in serum TNF-α concentration between migraineurs and controls, the levels of this proinflammatory cytokine correlated negatively with homocysteine and glucose concentrations in migraine patients. The link between homocysteine and TNF-α level was observed in hypertensive patients; however, such correlation in this particular group of patients was positive, while in our study we have found significant negative correlations. Thus, both of these factors creating inflammatory milieu are inversely related in migraine patients during the interictal period and may indicate monocytes’ insufficiency to produce TNF-α in reaction to increasing homocysteine levels.

Furthermore, serum TNF concentrations correlated positively with MIP-1, which is a CC chemokine involved in the early stages of inflammation, wound healing and hematopoiesis [40]. It has been shown to activate monocytes and basophils, it has chemotactic properties for T lymphocytes, eosinophils and monocytes [41,42]. MIP-1 induces the production of TNF-α in macrophages [41]. Thus, positive correlation between serum TNF-α concentrations and MIP-1 levels observed in our study indicates the role of this chemokine in monocytic stimulation in migraine patients during the interictal period. Serum MIP-1 levels correlated positively also with clinimetric measures of pain, and the MIP-1:CD14 ratio in monocyte fraction correlated negatively with MIGSEV-Pain scores, suggesting the possible role of this chemokine in the development of hyperalgesia in migraineurs and its depletion in monocytes. Serum MIP-1 concentrations in migraine patients are at the same levels as in controls. This is in contrast to results by Durate et al. who found that MIP-1α levels were higher among migraineurs than in controls [43]. One possible explanation for such difference between our study and the one by Durate et al. is that we only included patients with episodic migraine, while their population consisted of both, episodic and chronic migraine patients. Also, while our study group consisted of Caucasian patients only, the group investigated by Durate et al. was of multiple ethnicity. Since no other studies on the role of MIP-1 in migraine have been published, it remains to be established how MIP-1 is involved in migraine pathophysiology. In our study, MIP-1 correlated negatively with Lp(a) concentrations in migraine patients. Lipoprotein (a) is known to cause monocytes’ recruitment to the vessel wall, which results from the stimulation of endothelial cells [44,45]. The negative correlation between Lp(a) and MIP-1 level in migraineurs may be caused by endothelial dysfunction, which was shown [46] via the reduced number and functions of circulating endothelial progenitor cells in migraine patients.

## 3. Materials and Methods

Forty migraine patients (26 females, 14 males, mean age 39 ± 11 years) were included in the study. All patients fulfilled International Headache Society 2004 criteria for migraine diagnosis [47]. The study protocol was accepted by Ethic Committee of Poznan University of Medical Sciences (approval number 24/05, 6 January 2005). The studied cohort consisted of adult patients with episodic migraine. Consecutive migraine patients consulted in the Department of Neurology at Poznan University of Medical Sciences were included in the study. Migraineurs without aura and with aura participated in the study. The patients used only abortive treatment including triptans (sumatriptane, rizatriptane, zolmitriptane), ergot alkaloids, non-steroid anti-inflammatory drugs (NSAIDs) or analgesics (paracetamol, acetylsalicylic acid, ibuprofen, ketoprofen, diclofenac, naproxene, metamizole, tramadole). Patients under prophylactic migraine treatment were not included in the study. Written informed consent was obtained from all the participants. The study protocol was approved by the Internal Review Board at the Poznan University of Medical Sciences. To eliminate the effects of systemic disease, the following exclusion criteria were used: history of cardiovascular disease, hypertension (defined as systolic blood pressure exceeding 140 mm Hg or diastolic over 90 mm Hg), diabetes, hyperlipidemia, pregnancy, lactation, inflammation, allergy, psychiatric disorders, regular use of steroids or NSAIDs, any chronic pharmacotherapy. All subjects underwent full neurological examination and clinimetric evaluation with the use of Migraine Severity scale (MIGSEV) [48,49], Migraine Disability Assessment (MIDAS) [50], QVM (Qualité de Vie et Migraine) [49,51], VAS (Visual Analogue Scale) and VRS (Verbal Rating Scale). The scores of clinical scales reflected the evaluation of average intensity of attack features. Blood samples were taken no earlier than 4 days after migraine attack and/or administration of abortive treatment (triptans, ergot alkaloids, NSAIDS or analgesics).

**Figure 1:**
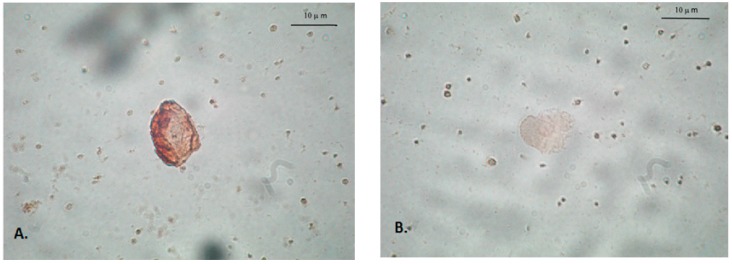
The expression of TNF-α in the monocytes of controls (**A**); and migraine patients (**B**).

Based on medical history, physical examination, and routine laboratory tests (see Table 1), none of the migraineurs or controls showed symptoms of any active or chronic disease. Thirty-nine healthy subjects (20 females, 19 males, mean age 37 ± 8 years) were used as controls. Healthy volunteers included in the control group were age-matched adult subjects, who did not experience any pain and were not treated chronically with any drugs or dietary supplements. Heparinized blood was used for monocyte separation. Following meglumineamidotrizoate-Ficoll centrifugation monocytes were isolated with the use of magnetic labeling system (MACS, MiltenyiBiotec GmbH, Bergisch Gladbach, Germany). May–Grunwald–Giemsa staining of obtained cells was performed to test the procedure. Monocytes underwent further immunostaining with the use of anti-TNF-α antibodies (Bender System GmbH, Vienna, Austria), see Figure 1A,B. The intensity of staining obtained as a result of the peroxidase-DAB reaction was estimated using ImageJ software (version 1.49d, freeware available at National Institute of Health USA, http://imagej.nih.gov/ij).).

Statistical analysis was performed with the use of the licensed MedCalc software version 16.8.4—64 bit. First, we have tested the distribution of results with the d’Agostino–Pearson test. Then, the results with Gaussian distribution were expressed as mean ± standard deviation (SD) and analyzed with the*t*-Student test. Variables with non-Gaussian distribution were presented as median and interquartile range, and tested with a non-parametric Mann–Whitney test. The correlations between variables were tested with univariate analysis and expressed as Spearman coefficient (rS).

## 4. Conclusions

Concluding, the abnormalities of TNF-α expression in monocytes, correlations of serum TNF-α and MIP-1 levels with clinimetric scores and increased circulating CD14 in migraine patients observed in the present study suggest the involvement of these factors that orchestrate components of the immune system during the interictal period. Understanding the role played by mediators of inflammation evaluated in our study may be promising for future therapeutic development.

## Figures and Tables

**Table 1:** Inflammation and autoimmunity markers in migraine patients and controls.

Parameter	Controls	Migraine Patients	*p* Value
ESR (mm/h)	7 ± 4	7 ± 5	*p* > 0.05
WBC (G/L)	6.4 ± 1.5	5.3 ± 1.5	*p* = 0.04 *
hsCRP (mg/dL)	0.75 (0.31–1.09)	0.42 (0.19–0.65)	*p* > 0.05
AAG (g/L)	0.66 ± 0.19	0.59 (0.54–0.64)	*p* > 0.05
IgE (IU/mL)	27.7 (20.2–53.6)	32.4 (20.2–59.3)	*p* > 0.05
Anticardiolipin antibodies (IgM) (U/mL)	1.10 (0.70–1.80)	1.30 (0.80–1.60)	*p* > 0.05
Anticardiolipin antibodies (IgG) (U/mL)	0.40 (0.30–0.60)	0.60 (0.40–1.90)	*p* = 0.04 *
Anti-nuclear antibodies	Negative in all patients	Negative in all subjects	
Anti—dsDNA	Negative in all patients	Negative in all subjects	
Anti-MPO p-ANCA	Negative in all patients	Negative in all subjects	
Anti-Pr3 c-ANCA	Negative in all patients	Negative in all subjects	
ASMA	Negative in all patients	Negative in all patients	
APCA	Negative in all patients	Negative in all patients	
AMA	Negative in all patients	Negative in all patients	
HMA	Negative in all patients	Negative in all patients	

ESR—erythrocyte sedimentation rate, WBC—white blood cells, hsCRP—high sensitivity CRP, AAG—alpha 1-acid glycoprotein, Anti-dsDNA—anti-double stranded DNA, anti-MPO/pANCA—anti-myeloperoxidase/perinuclear pattern anti-neutrophil cytoplasm autoantibodies, Anti-Pr3/c-ANCA—anti-proteinase 3/cytoplasmic pattern anti-neutrophil cytoplasm autoantibodies, ASMA—anti-smooth muscle antibodies, APCA—anti-parietal cell antibodies, AMA—antimitochondrial antibodies, HMA—hepatocyte membrane antibodies, * statistically significant. TNF-α and MIP-1 levels were estimated by means of ELISA (R&D Systems and Bender MedSystems, respectively) in the monocytes’ fraction and serum. The expression of both was depicted as a ratio to CD14 (TNF-α:CD14, MIP-1:CD14). CD14 was analyzed by means of the ELISA (R&D Systems) technique in the monocytes’ fraction and serum.

**Table 2:** Body mass index (BMI), glucose, plasma lipids and homocysteine concentrations in migraine patients and controls.

Parameter	Controls	Migraine Patients	*p* Value
BMI	22 ± 3	23 ± 1	*p* > 0.05
Glucose (mg/dL)	64 ± 17	63 ± 12	*p* > 0.05
Total cholesterol(mg/dL)	190 ± 33	210 ± 38	*p* > 0.05
HDL-cholesterol(mg/dL)	64 ± 20	63 ± 14	*p* > 0.05
LDL-cholesterol(mg/dL)	110 ± 39	131 ± 36	*p* > 0.05
TAG(mg/dL)	94 ± 50	83 ± 30	*p* > 0.05
Lp(a) (g/L)	0.08(0.03–0.3)	0.10(0.04–0.20)	*p* > 0.05
Homocysteine(mmol/L)	13.6 ± 4.5	15.0 ± 6.5	*p* > 0.05

HDL—high density lipoprotein, LDL—low density lipoprotein, TAG—triacylglycerols, Lp(a)—lipoprotein (a).

**Table 3:** Serum MIP-1, TNF-α, CD14 and MIP-1:CD14 and TNF-α:CD14 in monocyte fraction in migraine patients and controls.

Serum Parameter	Controls	Migraineurs	*p* Value
MIP-1 (pg/mL)			
median	0	0	
(interquartile range)	(0.0–0.0)	(0.0–0.0)	0.99
TNF-α (pg/mL)			
median	1.108	0.494	
(interquartile range)	(0.617–3.012)	(0.494–1.292)	0.11
CD14 (pg/mL)			
median	2635	4118	
(interquartile range)	(2085–3870)	(3174–5660)	0.008 *
MIP-1:CD14			
median	0	0.037	
(interquartile range)	(0.0–0.137)	(0.0–0.191)	0.45
TNF-α:CD14			
median	0.005	0.005	
(interquartile range)	(0.003–0.012)	(0.0–0.014)	0.85

* statistically significant.

**Table 4:** Serum levels of MIP-1, TNF-α and CD14, TNF-α:CD14 and MIP-1:CD14 ratios in monocytes in migraine patients with and without aura.

Serum Parameter	Migraineurs with Aura	Migraineurs without Aura	*p* Value
MIP-1 (pg/mL)			
median	0	0	
(interquartile range)	(0.0–37.77)	(0.0–0.0)	*p* = 0.87
TNF-α (pg/mL)			
median	0.49	0.49	
(interquartile range)	(0.49–2.24)	(0.49–0.92)	*p* = 0.96
CD14 (pg/mL)			
median	4361	4117	
(interquartile range)	(3314–5438)	(3119–5725)	*p* = 0.98
MIP-1:CD14			
median	0.06	0.01	
(interquartile range)	(0.0–0.50)	(0.0–0.13)	*p* = 0.62
TNF-α:CD14			
median	0.01	0	
(interquartile range)	(0.0–0.3)	(0.0–0.01)	*p* = 0.64

**Table 5:** Serum levels of MIP-1, TNF-α and CD14, TNF-α:CD14 and MIP-1:CD14 ratios in monocytes in migraine patients using and not using NSAIDs as abortive treatment.

Serum Parameter	Migraineurs Using NSAIDs	Migraineurs without NSAIDs	*p* Value
MIP-1 (pg/mL)			
median	0	0	
(interquartile range)	(0.0–0.0)	(0.0–281.0)	*p* = 0.27
TNF-α (pg/mL)			
median	0.49	0.92	
(interquartile range)	(0.49–0.80)	(0.49–4.12)	*p* = 0.14
CD14 (pg/mL)			
median	4088	4997	
(interquartile range)	(3202–5725)	(2147–5660)	*p* = 0.69
MIP-1:CD14			
median	0.06	0.01	
(interquartile range)	(0.0–0.16)	(0.0–0.209)	*p* = 0.97
TNF-α:CD14			
median	0.01	0	
(interquartile range)	(0.0–0.02)	(0.02–0.08)	*p* = 0.59

**Table 6:** Correlations between analyzed cytokines, chemokines, lipoprotein (a) and homocysteine in migraine patients.

Serum Parameter	Lp(a)	Hcy	Serum MIP-1	TNF-α:CD14 in Monocytes
Serum MIP-1	rS = −0.5218, *p* = 0.007	NS	x	NS
Serum TNF	rS = −0.505, *p* = 0.01	rS = −0.4334, *p* = 0.03	rS = 0.8218, *p* < 0.001	NS
MIP-1:CD14 in monocytes	NS	NS	NS	rS = 0.7263, *p* < 0.001

NS—not significant; Lp(a)—lipoprotein (a); Hcy—homocysteine; rS—Spearman coefficient

**Table 7:** Correlations between analyzed cytokines, chemokines, hsCRP and clinimetric measures in migraine patients.

Serum Parameter	VAS	VRS	MigSev—Pain	Years of Migraine Duration
hsCRP	rS = 0.300, *p* = 0.0361	rS = 0.5584, *p* = 0.01	NS	NS
Serum CD14	NS	NS	NS	rS = 0.293, *p* = 0.042
Serum MIP-1	NS	rS = 0.4683, *p* = 0.04	NS	NS
MIP-1:CD14 in monocytes	rS = −0.5051, *p* = 0.02	NS	rS = −0.5064, *p* = 0.01	rS = −0.452, *p* = 0.0232

NS—not significant; rS—Spearman coefficient

## References

[1] Mortimer M.J., Kay J., Gawkrodger D.J., Jaron A., Barker D.C. (1993). The prevalence of headache and migraine in atopic children: An epidemiological study in general practice. Headache.

[2] Covelli V., Massari F., Conrotto L., D’Andrea I.L., Maffione A.B., Jirrillo E., Buscaino G.A. (1993). Demonstration of an elevated frequency of infectious events in patients with migraine without aura: A correlation with their altered immune status. J. Immunol. Immunopharmacol..

[3] Pinessi L., Savi L., Pellicano R., Rainero I., Valfrè W., Gentile S., Cossotto D., Rizzetto M., Ponzetto A. (2000). Chronic Helicobacter pylori infection and migraine: A case-control study. Headache.

[4] Cohen S.I., Blau J.N. (2003). Lifelong migraine aura without headache: Change of pattern with upper respiratory infection. J. R. Soc. Med..

[5] Lord G.D., Duckworth J.W., Charlesworth J.A. (1977). Complement activation in migraine. Lancet.

[6] Lord G.D., Duckworth J.W. (1977). Immunoglobulin and complement studies in migraine. Headache.

[7] Covelli V., Maffione A.B., Munno I., Jirillo E. (1990). Alterations of nonspecific immunity in patients with common migraine. J. Clin. Lab. Anal..

[8] Leone M., Biffi M., Leoni F., Bussone G. (1994). Leukocyte subsets and cortisol serum levels in patients with migraine without aura and chronic tension-type headache. Cephalalgia.

[9] Visintini D., Trabattoni G., Manzoni G.C., Lechi A., Bortone L., Behan P.O. (1986). Immunological studies in cluster headache and migraine. Headache.

[10] Gallai V., Sarchielli P., Floridi A., Franceschini M., Trequattrini A., Firenze C. (1994). Monocyte function in migraine patients with and without aura. Headache Q..

[11] Gilman-Sachs A., Robbins L., Baum L. (1989). Flow cytometric analysis of lymphocyte subsets in peripheral blood of chronic headache patients. Headache.

[12] Martelletti P., Sutherland J., Anastasi E., Di Mario U., Giacovazzo M. (1989). Evidence for an immune-mediated mechanism in food-induced migraine from a study on activated T-cells, IgG4 subclass, anti-IgG antibodies and circulating immune complexes. Headache.

[13] Covelli V., Munno I., Pellegrino N.M., di Venere A., Jirillo E., Buscaino G.A. (1990). Exaggerated spontaneous release of tumor necrosis factor-α/cachectin in patients with migraine without aura. Acta Neurol. (Napoli).

[14] Covelli V., Munno I., Pellegrino N.M., Altamura M., Decandia P., Marcuccio C., di Venere A., Jirillo E. (1991). Are TNF-α and IL-1 beta relevant in the pathogenesis of migraine without aura?. Acta Neurol. (Napoli).

[15] Martelletti P., Stirparo G., Rinaldi C., Frati L., Giacovazzo M. (1993). Disruption of the immunopeptidergic network in dietary migraine. Headache.

[16] Shimomura T., Araga S., Kowa H., Takahashi K. (1992). Immunoglobulin kappa/lambda ratios in migraine and tension-type headache. Jpn. J. Psych. Neurol.

[17] Jerzmanowski A., Klimek A. (1983). Immunoglobulins and complement in migraine. Cephalalgia.

[18] Mosnaim A.D., Kulaga H., Adams A.J., Wolf M.E., Puente J., Freitag F., Diamond S. (1998). Flow cytometric analysis of lymphocyte subsets in migraine patients during and outside of an acute headache attack. Cephalalgia.

[19] Battistella P.A., Bordin A., Cernetti R., Broetto S., Corrà S., Piva E., Plebani M. (1996). Beta endorphin in plasma and monocytes in juvenile headache. Headache.

[20] Shimomura T., Araga S., Esumi E., Takahashi K. (1991). Decreased serum interleukin-2 level in patients with chronic headache. Headache.

[21] Williamson D.J., Hargreaves R.J. (2001). Neurogenic inflammation in the context of migraine. Microsc. Res. Tech..

[22] Thalakoti S., Patil V.V., Damodaram S., Vause C.V., Langford L.E., Freeman S.E., Durham P.L. (2007). Neuron-glia signaling in trigeminal ganglion: Implications for migraine pathology. Headache.

[23] Oprée A., Kress M. (2000). Involvement of the proinflammatory cytokines tumor necrosis factor-α, IL-1β, and IL-6 but not IL-8 in the development of heat hyperalgesia: Effects on heat-evoked calcitonin gene-related peptide release from rat skin. J. Neurosci..

[24] Reuter U., Bolay H., Jansen-Olesen I., Chiarugi A., Sanchez del Rio M., Letourneau R., Theoharides T.C., Waeber C., Moskowitz M.A. (2001). Delayed inflammation in rat meninges: Implications for migraine pathophysiology. Brain.

[25] Lassen L.H., Ashina M., Christiansen I., Ulrich V., Olesen J. (1997). Nitric oxide synthase inhibition in migraine. Lancet.

[26] Durham P.L. (2006). Emerging Neural Theories of Migraine pathogenesis: Calcitonin gene-related peptide (CGRP) and migraine. Headache.

[27] Theoharides T.C., Spanos C., Pang X., Alferes L., Ligris K., Letourneau R. (1995). Stress-induced intracranial mast cell degranulation: A corticotropinreleasing hormone-mediated effect. Endocrinology.

[28] Rozniecki J.J., Dimitriadou V., Lambracht-Hall M., Pang X., Theoharides T.C. (1999). Morphological and functional demonstration of rat dura mater mast cell—Neuron interactions in vitro and in vivo. Brain Res.

[29] Sarchielli P., Floridi A., Mamcini M.L., Rossi C., Coppola F., Baldi A., Pini L.A., Calabresi P. (2006). NF-κB activity and iNOS expression in monocytes from internal jugular blood of migraine without aura patients during attacks. Cephalalgia.

[30] Krumholz W., Szalay G., Ogal H., Menges T. (2000). Effect of migraine medications on monocyte chemotaxis. Anaesthesiol. Reanim..

[31] Martelletti P., Stirparo G., Rinaldi C., Fusco B.M. (1994). Function of the peripheral serotoninergic pathways in migraine: A proposal for an experimental model. Cephalalgia.

[32] Stirparo G., Zicari A., Favilla M., Lipari M., Martelletti P. (2000). Linked activation of nitric oxide synthase and cyclooxygenase in peripheral monocytes of asymptomatic migraine without aura patients. Cephalalgia.

[33] Hailman E., Vasselon T., Kelley M., Busse L.A., Hu M.C., Lichenstein H.S., Detmers P.A., Wright S.D. (1996). Stimulation of macrophages and neutrophils by complexes of lipopolysaccharide and soluble CD14. J. Immunol..

[34] Bazil V., Horejsi V., Baudys M., Kristofová H., Strominger J.L., Kostka W., Hilgert I. (1986). Biochemical characterization of a soluble form of the 53-kDa monocyte surface antigen. Eur. J. Immunol..

[35] Rietschel E.T., Schletter J., Weidemann B., El-Samalouti V., Mattern T., Zähringer U., Seydel U., Brade H., Flad H.D., Kusumoto S. (1998). Lipopolysaccharide and peptidoglycan: CD14-dependent bacterial inducers of inflammation. Microb. Drug Resist..

[36] Kitchens R.L., Thompson P.A., Viriyakosol S., O’Keefe G.E., Munford R.S. (2001). Plasma CD14 decreases monocyte responses to LPS by transferring cell-bound LPS to plasma lipoproteins. J. Clin. Investig..

[37] Sugiyama T., Wright S.D. (2001). Soluble CD14 mediates efflux of phospholipids from cells. J. Immunol..

[38] Bazil V., Strominger J.L. (1991). Shedding as a mechanism of down-modulation of CD14 on stimulated human monocytes. J. Immunol..

[39] Cauwels A., Frei K., Sansano S., Fearns C., Ulevitch R., Zimmerli W., Landmann R. (1999). The origin and function of soluble CD14 in experimental bacterial meningitis. J. Immunol..

[40] Davatelis G., Tekamp-Olson P., Wolpe S.D., Hermsen K., Luedke C., Gallegos C., Coit D., Merryweather J., Cerami A. (1988). Cloning and characterization of a cDNA for murine macrophage inflammatory protein (MIP), a novel monokine with inflammatory and chemokinetic properties. J. Exp. Med..

[41] Fahey T.J., Tracey K.J., Tekamp-Olson P., Cousens L.S., Jones W.G., Shires G.T., Cerami A., Sherry B. (1992). Macrophage inflammatory protein 1 modulates macrophage function. J. Immunol..

[42] Li H., Sim T.C., Grant J.A., Alam R. (1996). The production of macrophage inflammatory protein-1 α by human basophils. J. Immunol..

[43] Durate H., Teixeira A., Rocha N.P., Domingues R.B. (2015). Increased interictal serum levels of CXCL8/IL-8 and CCL3/MIP-1α in migraine. Neurol. Sci..

[44] Poon M., Zhang X., Dunsky K., Taubman M.B., Harpel P.C. (1997). Apolipoprotein(a) is a human vascular endothelial cell agonist: Studies on the induction in endothelial cells of monocyte chemotactic factor activity. Clin. Genet..

[45] Haque N.S., Zhang X., French D.L., Li J., Poon M., Fallon J.T., Gabel B.R., Taubman M.B., Koschinsky M., Harpel P.C. (2000). CC chemokine I-309 is the principal monocyte chemoattractant induced by apolipoprotein(a) in human vascular endothelial cells. Circulation.

[46] Lee S., Chu K., Jung K. (2008). Decreased number and function of endothelial progenitor cells in patients with migraine. Neurology.

[47] Headache Classification Subcommittee of the International Headache Society (2004). The International Classification of Headache Disorders: 2nd edition. Cephalalgia.

[48] El Hasnaoui A., Vray M., Richard A., Nachit-Ouinekh F., Boureau F., MIGSEV Group (2003). Assessing the severity of migraine: Development of the MIGSEV scale. Headache.

[49] El Hasnaoui A., Vray M., Blin P., Nachit-Ouinekh F., Boureau F., The HEMISHERE Study Group (2004). Assessment of migraine severity using the MIGSEV scale: Relationship to migraine features and quality of life. Cephalalgia.

[50] Stewart W.F., Lipton R.B., Dowson A.J., Sawyer J. (2001). Development and testing of the Migraine Disability Assessment (MIDAS) Questionnaire to assess headache-related Disability. Neurology.

[51] Richard A., Henry P., Chazot G., Massiou H., Tison S., Marconnet R., Chicoye A., d’Allens H. (1993). Quality of life and migraine. Validation of the QVM questionnaire in hospital consultation and in generalmedicine. Therapie.

